# Drivers of species richness and beta diversity of fishes in an Afrotropical intermittent river system

**DOI:** 10.1002/ece3.9659

**Published:** 2022-12-28

**Authors:** Ray C. Schmidt, Taylor Woods, Wanja D. Nyingi

**Affiliations:** ^1^ Biology Department Randolph‐Macon College Ashland Virginia USA; ^2^ Division of Fishes National Museum of Natural History Washington District of Columbia USA; ^3^ Department of Ecology and Evolutionary Biology The University of Tennessee Knoxville Tennessee USA; ^4^ Ichthyology Section National Museums of Kenya Nairobi Kenya

**Keywords:** beta diversity, biodiversity conservation, nestedness, water extraction

## Abstract

Tropical freshwater ecosystems are some of the most threatened systems yet remain understudied relative to temperate systems. Here, we look at the drivers of community structure of fishes in a tropical and intermittent system in central Kenya. We conducted monthly samples within the upper Northern Ewaso Ng'iro to assess variation in community composition and abiotic characteristics. We analyzed species richness along the longitudinal gradient, computed beta diversity within the system, relative contributions of each site, and partitioned beta diversity metrics into nestedness and turnover components. We found that, similar to temperate intermittent systems, species richness varied along the longitudinal gradient, nestedness contributions to beta diversity exceeded those of turnover, and environmental and spatial variables determined patterns of beta diversity. Sites at the highest and lowest ends of the species richness gradient showed the highest contributions to beta diversity, suggesting sites important for preservation or restoration initiatives, respectively. With ongoing water extraction and conflict over resources throughout the region, this study highlights the need for further investigations of the effects of multiple stressors on biodiversity patterns and ecosystem functioning in tropical stream communities.

## Graphical abstract

Here, we look at the beta diversity and drivers of species richness in fishes in the upper Northern Ewaso Ng'iro River in Kenya. We found nestedness contributions to beta diversity exceeded those of turnover. Sites at the highest and lowest ends of the species richness gradient showed the highest contributions to beta diversity, suggesting sites important for preservation or restoration initiatives, respectively.

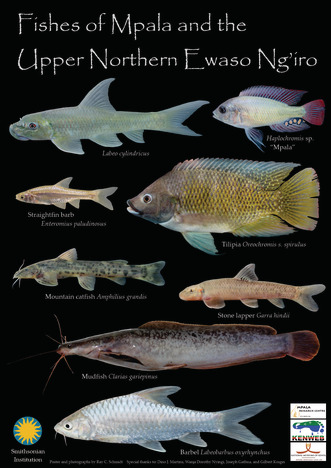

## 1 INTRODUCTION

Freshwater ecosystems are some of the most threatened and least understood systems globally (Darwall et al., 2018). Roughly 70% of the vertebrates that are found in freshwater ecosystems are under threat of extinction, and the effects of the loss of biodiversity in these systems remain unknown (Darwall et al., 2018). Tropical montane rivers are home to incredible levels of biodiversity, provide livelihoods to hundreds of millions of people, and are seemingly at the most risk from the effects of global climate change yet remained largely understudied (Encalada et al., 2019). Recent efforts elucidating some of the dynamics of temperate freshwater communities can now be applied to these comparatively understudied tropical ecosystems.

Understanding factors that determine patterns in community structure through space and time is important for biodiversity conservation. One aspect of biodiversity that is important to consider is variation in species community composition attributed to relationships between local (alpha) and regional (gamma) diversity (i.e., beta diversity; Whittaker, 1960). Beta diversity analyses are valuable when assessing changes in composition along abiotic gradients (e.g., altitudinal and latitudinal gradients) and may be useful to explain the processes driving larger‐scale biodiversity patterns (Kraft et al., 2011; Qian et al., 2005, 2009; Tang et al., 2012).

Beta diversity can be partitioned into nestedness and turnover components. Nestedness occurs when species‐poor communities are subsets of speciose communities and is usually driven by stochastic processes (Baselga, 2010; Gaston & Blackburn, 2000), whereas turnover occurs when changes in species composition occur via the replacement of species as ecological or environmental conditions change (Baselga, 2010; Qian et al., 2005). Generally, environmental and spatial factors are thought to be the two main classes of factors to drive patterns of beta diversity and its components (Duivenvoorden et al., 2002; Eros et al., 2012; Heino et al., 2012; Menegotto et al., 2019). In intermittent systems, it is generally hypothesized that the drying of the stream will create longitudinally nested communities because these harsh environmental conditions could lead to restricted dispersals and extinctions (Larned et al., 2010).

Environmental factors that affect beta diversity may vary depending on the ecosystem considered but generally reflect gradients that likely physiologically constrain organisms' distributions (e.g., elevation, Tang et al., 2012). Spatial factors (e.g., geographic distance) are also important due to widely observed relationships between community dissimilarity and geographic distance (e.g., distance decay; Soininen et al., 2007). The contribution of a single site to overall patterns in beta diversity in a system can be described using local contributions to beta diversity (LCBD; Legendre & De Cáceres, 2013). Increasing values of LCBD indicate greater individual contribution of a site to driving the overall patterns of beta diversity. That is, sites that have unique combinations of species are likely to have higher LCBD values and sites that are degraded or species poor are also likely to have higher LCBD values (Legendre & De Cáceres, 2013). In looking at sites along an elevational gradient a U‐shaped LCDB curve is usually produced suggesting that the higher and lower elevation sites both have higher LCBD values than sites in the middle of the gradient (Wang et al., 2020). From a conservation perspective this metric can indicate sites with high preservation priority, or degraded sites with priority for restoration efforts (Kong et al., 2017).

Communities of stream fishes are particularly good candidates for beta diversity analyses because the dendritic structure of stream systems results in environmental and spatial gradients upon which to analyze changes in species composition (Heino et al., 2015; Rogosch & Olden, 2019; Vannote et al., 1980). However, much of the literature on stream beta diversity derives from perennial streams in temperate and subtropical regions (Griffiths, 2017; Heino, 2011; Matthews & Marsh‐Matthews, 2016, 2017; Rolls et al., 2016; Tornés & Ruhí, 2013; Zbinden & Matthews, 2017). Comparatively, fewer studies of beta diversity exist for tropical intermittent systems, and especially Afrotropical riverine communities of fishes. These studies, largely focused on perennial systems in the Lake Victoria Basin, provided invaluable information on these systems and found diversity generally increased along the longitudinal gradient and changes to land use affect community composition (Achieng et al., 2020; Fugère et al., 2016; Masese et al., 2020). Considering ongoing environmental change, investigating beta diversity patterns in these tropical intermittent rivers allows a better understanding of the dynamics in these systems and will help develop management plans for these critical and diverse systems (Encalada et al., 2019; Myers et al., 2000).

Here, we examine longitudinal variation in community composition of fishes within an intermittent Afrotropical montane river system, the Northern Ewaso Ng'iro, in central Kenya. Ongoing water extraction in the upper reaches of the river, increasing land‐use conversion to agriculture, and the effects of climate change threaten the persistence of stream biota and the human populations that rely on this system for their survival (Ngigi et al., 2008). These activities are also contributing to an increase in human‐wildlife and human‐human conflict in the region (Bond, 2014; Gichuki, 2002; Lesrima, 2019). Additionally, a large‐scale impoundment is approved for construction on the main river channel with the effects on freshwater communities and downstream users not yet known. Our aim was to clarify the patterns and drivers of spatial patterns of biodiversity of fishes in this basin, with a focus on testing hypotheses from intermittent systems. We hypothesize: (1) that species richness increases along the longitudinal river gradient from the headwater tributaries to mainstem river sites, (2) the longitudinal species richness gradient will drive LCBD patterns, with greatest LCBD observed at the sites at either end of the gradient (lowest and highest species richness values) resulting in a U‐shaped pattern, and (3) beta diversity partitioning in this system will indicate the nestedness component exceeds that of the turnover component, as has been hypothesized for intermittent stream systems (Larned et al., 2010). Additionally, we test for effects of environment and spatial predictors on driving spatial patterns in beta diversity, nestedness, and turnover in this system.

## 2 METHODS

### 2.1 Study system and sites

**FIGURE 1:**
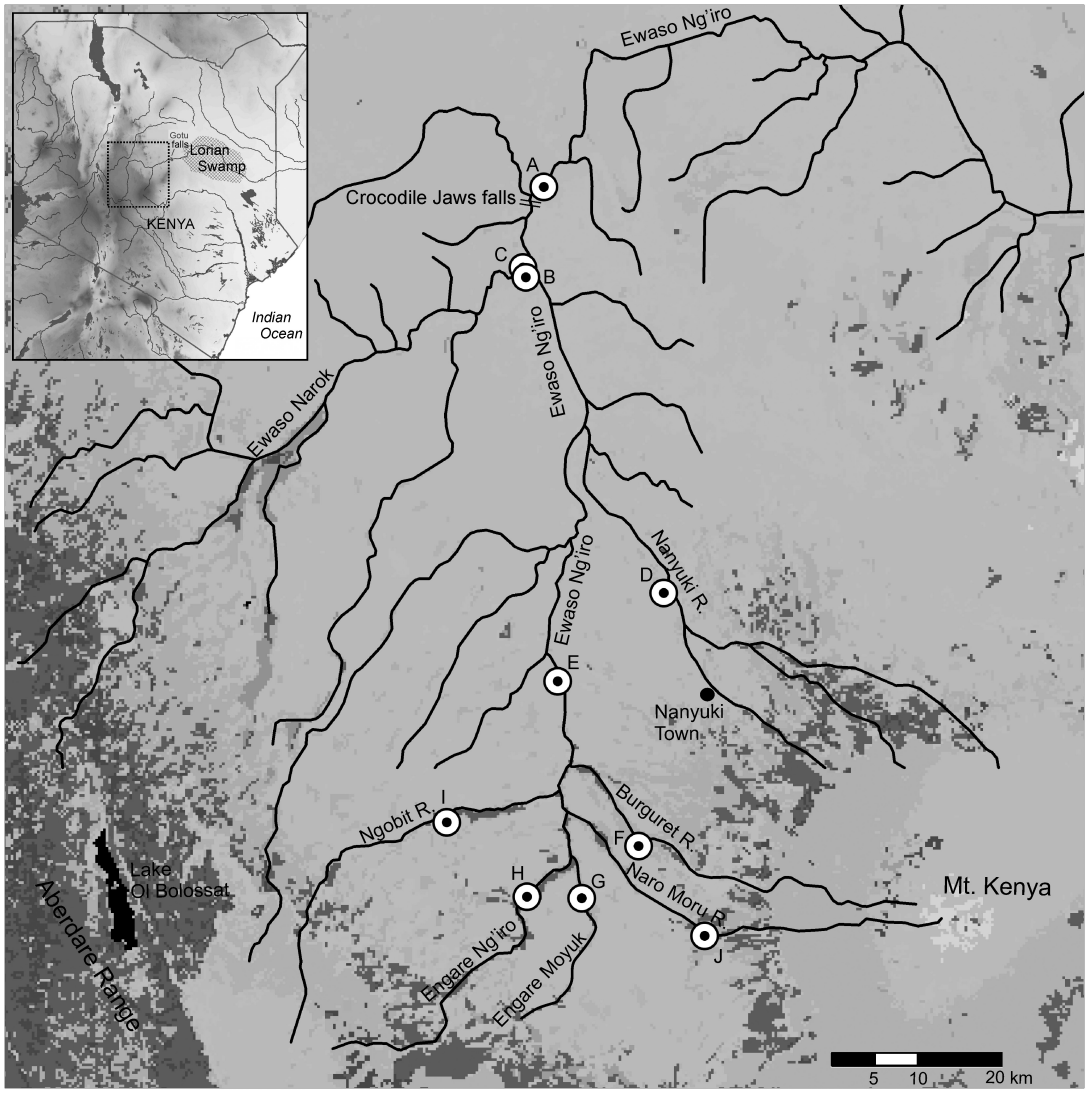
Locations of sampling sites (*n* = 10) within the northern Ewaso Ng'iro basin, Central Kenya, illustrating land use practices in the study region (dark grays are cropland and mosaic cropland), major topographic features and urban areas. The inset map shows the study region within Kenya, illustrating elevation throughout the country.

The Northern Ewaso Ng'iro in Kenya is unique in the breadth of habitat, land‐use practices, and the varied users that depend on the system for their livelihood and survival. The Northern Ewaso Ng'iro's headwaters arise on the well‐watered slopes of Mt Kenya and the Aberdare Mountains (Figure 1). From these headwater streams, the system passes through regions with large‐scale floriculture and smaller‐scale agriculture where much of the available water is extracted for agricultural and domestic purposes (Ngigi et al., 2008). In recent years the rates of extraction have exceeded the amount of surface flows even during the wet periods (Gichuki, 2002). These waters then flow through semi‐arid rangeland and are critical for the pastoralist communities in the region. The Northern Ewaso Ng'iro goes through the chutes at Crocodile Jaws, site for the approved mega dam, shortly downstream of the confluence with the Ewaso Narok. The river then flows, intermittently, downstream to Gotu Falls (formerly known as Chanler's Falls), an important biogeographic barrier (Schmidt et al., 2014, 2015), and on toward the Lorian swamp. Finally, these waters, renewed by the freshwater Gotu and Buffalo Springs, end in the endorheic Lorain Swamp and help recharge the Merti Aquifer. The Lorain Swamp also serves as an important source of rangeland during the dry seasons for pastoralist communities from the frontier lands in northeastern Kenya and western Somalia (De Leeuw et al., 2012). Increased water extraction in the upper reaches of the drainage have reduced the flows of water downstream leading to conflict between pastoralists and upstream users and increased instances of human–wildlife conflict throughout the drainage (Bond, 2014; Fox, 2018; Gichuki, 2002).

### 2.2 Data collection

#### 2.2.1 Field data

Ten localities within the upper Northern Ewaso Ng'iro were sampled monthly from October 2015 through October 2016 (Figure 1). These sites ranged from smaller headwater streams, the main channels of the N. Ewaso Ng'iro and Ewaso Narok upstream of their confluence, and below Crocodile Jaws on the N. Ewaso Ng'iro downstream of the confluence with the Ewaso Narok at Sabuk Lodge (Table S1). Sampling at certain locations did not occur during months when high water made collecting impossible or unsafe.

Fishes were collected at each locality along 100–500 m stretches, depending on the stream width, and sampling all habitat types present. We sampled for species presence and relative abundance with single‐pass electrofishing (SAMUS 725 M) followed by seining (10 ft seines 3/16‐inch mesh) (Pusey et al., 1998) for 60–90 minutes at each locality. The single‐pass electrofishing and seining method is an effective way to assess the community of fishes especially in streams with relatively low species richness (Patton et al., 2000; Reid et al., 2009; Reynolds et al., 2003). Specimens were either collected and fixed in 10% formalin and later preserved in 75% ethanol or recorded and released. For some common and abundant species, not all specimens collected were kept for long‐term storage. All collections occurred under the supervision of staff from the National Museums of Kenya and were permitted by the National Commission for Science Technology & Innovation (Permit No: NACOSTI/P/15/7525/7403 & P/16/7525/13404 to RCS). All collected and preserved specimens were identified to species with published guides (Nyingi, 2016; Seegers et al., 2003) and deposited in the National Museums of Kenya Ichthyology Section in Nairobi.

At each locality, water temperature, dissolved oxygen, pH, and conductivity were collected with a water quality probe (YSI model 85; Yellow Springs Instruments). Percentage of riparian and aquatic vegetation, canopy cover, bank slope, percentage of riffle, run, and pool habitats, and erosion or other disturbance along the study site were also noted. All measurements and collection data associated with this study are available on the Freshwater Biodiversity Data Portal (Schmidt et al., 2020).

#### 2.2.2 Beta diversity metrics

We aggregated species lists from each sampling event into a presence–absence matrix (species × sampling event). We calculated species richness as the sum of species observed at a site across all sampling events and calculated beta diversity between sites (spatial beta diversity) for all possible pairwise combinations. Next, we decomposed total beta diversity into its nestedness and turnover components following Baselga (2010). According to this framework, pairwise Sorenson beta diversity (*β*_sor_), a measure of compositional dissimilarity, is partitioned into two additive components, nestedness (*β*_nes_) and turnover (*β*_sim_), such that *β*_sor_ = *β*_nes_ + *β*_sim_ (Baselga, 2010). Values for all measures range from 0 to 1, where values of 0 indicate an absence of beta diversity (identical species composition), and values of 1 indicate maximal beta diversity (dissimilar species composition). We used the package “betapart” (Baselga et al., 2018) in R (R Core Team, 2019) to calculate beta diversity metrics. We calculated the average beta diversity values between site pairs across sampling events for each component. We also assessed temporal variation in species composition among samples within a site and calculated temporal beta diversity between consecutive samples per site and used the average temporal beta diversity to describe variability in species composition among months (higher values indicate more variable species composition).

We calculated the local contributions to beta diversity (LCBD) to estimate the contribution of each site to spatial *β*_sor_, *β*_nes_, and *β*_sim_. Higher LCBD values indicate a site may represent species composition that is important for overall beta diversity within the system. We calculated LCBD for each site based on square root transformed distance matrices for each spatial beta diversity component. LCBD values were calculated in R using the package “adespatial” (Dray et al., 2020).

#### 2.2.3 Predictor variables

To characterize environmental factors that may drive patterns in beta diversity, we calculated the mean DO, percentage of pool habitat, and pH; and the mean and standard deviation of temperature across sampling events at each site. We also collected additional information on climate and land‐use at each site. We used the Climate Prediction and Applications Centre 2015 Kenya Land Cover Map (IGAD Climate Predictions and Applications Centre, 2015) and quantified land‐use as the percentages of land‐use/land cover categories within a 200‐meter buffer of each site sampled. The mean and standard deviation of monthly precipitation from 2015 to 2016 were collected at each site from the Global Precipitation Climatology Centre (Schneider et al., 2018).

To assess potential spatial influences on beta diversity in the system, we quantified the river distance between each site and the confluence of the Ewaso Narok and Ewaso Ng'iro Rivers. The confluence of this system contains the highest diversity and therefore represents the potential source pool from which species would disperse. River distances were recorded by manually tracing distances along the stream network in a Geographic Information System (ArcMap; ESRI, 2011). We also included the latitude and longitude of each site within the set of spatial predictor variables (Table S2).

### 2.3 Data analysis

#### 2.3.1 Alpha diversity

To test our prediction that species richness increases from headwater tributaries to the mainstem river, we used simple linear regression to model the relationship between species richness and distance to the confluence of the N. Ewaso Ng'iro and Narok Rivers. Distance to the confluence was our only predictor variable to prevent model overfitting. Both variables were standardized (mean = 0, SD = 1) prior to modeling. Akaike's Information Criterion (AIC) assessed whether the model including distance to confluence as a predictor was a better‐fit model than an intercept‐only (null) model. We inspected residual plots from the best‐fit model (QQ‐plots, residual vs. fitted values) and found no evidence of nonlinearity or heteroskedasticity in model residuals.

#### 2.3.2 Local contributions to beta diversity

To test our hypothesis that local contributions to beta diversity exhibit a U‐shaped relationship with species richness, we used regression models (*n* = 3) to model LCBD indices as a function of species richness. All LCBD values and species richness were standardized and we used AIC scores to assess whether a quadratic term for species richness provided a better fit compared to a linear model.

#### 2.3.3 Beta diversity

To test our hypothesis that the nestedness component of beta diversity exceeded that of turnover we used Wilcoxon Signed Rank tests to compare the distribution of nestedness to turnover components in the R package “stats” (R Core Team, 2019). To identify potential environmental and spatial drivers of beta diversity, we applied distance‐based redundancy analysis (db‐RDA; Legendre & Andersson, 1999). Prior to db‐RDA modeling, we reduced environmental predictors to a set of explanatory variables that were not highly correlated (|*r*| < .80). The environmental factors included were as follows: the mean and standard deviation of water temperature, mean dissolved oxygen, pH, and percentage of pool habitat, and the percentage of crop and shrub land cover. We applied *ln*(_*x + 1*_) transformations to all percentages and standardized all environmental and spatial predictors (Underwood, 1997). We performed db‐RDA on square root transformed distance matrices (*n* = 3; average spatial *β*_sor_, *β*_nes_, and *β*_sim_) as the response and the environmental and spatial variables, separately, as predictor variables (*n* = 6 db‐RDA models total).

For each db‐RDA model, we identified predictors that explained variation in the beta diversity component using a forward selection procedure with 999 permutations and stopping criteria based on significance of explanatory factors and comparison of adjusted *R* ^2^ of candidate models to the global (all predictors included) model (Blanchet et al., 2008). Following model selection, we performed an *F*‐test to test the significance (alpha = 0.05) of the reduced model. Next, we applied variation partitioning on the reduced models to determine the proportion of variance in spatial beta diversity explained by environmental versus spatial predictors. We used the R package “vegan” (Oksanen et al., 2019) to conduct db‐RDA and variation partitioning analyses.

## 3 RESULTS

**FIGURE 2:**
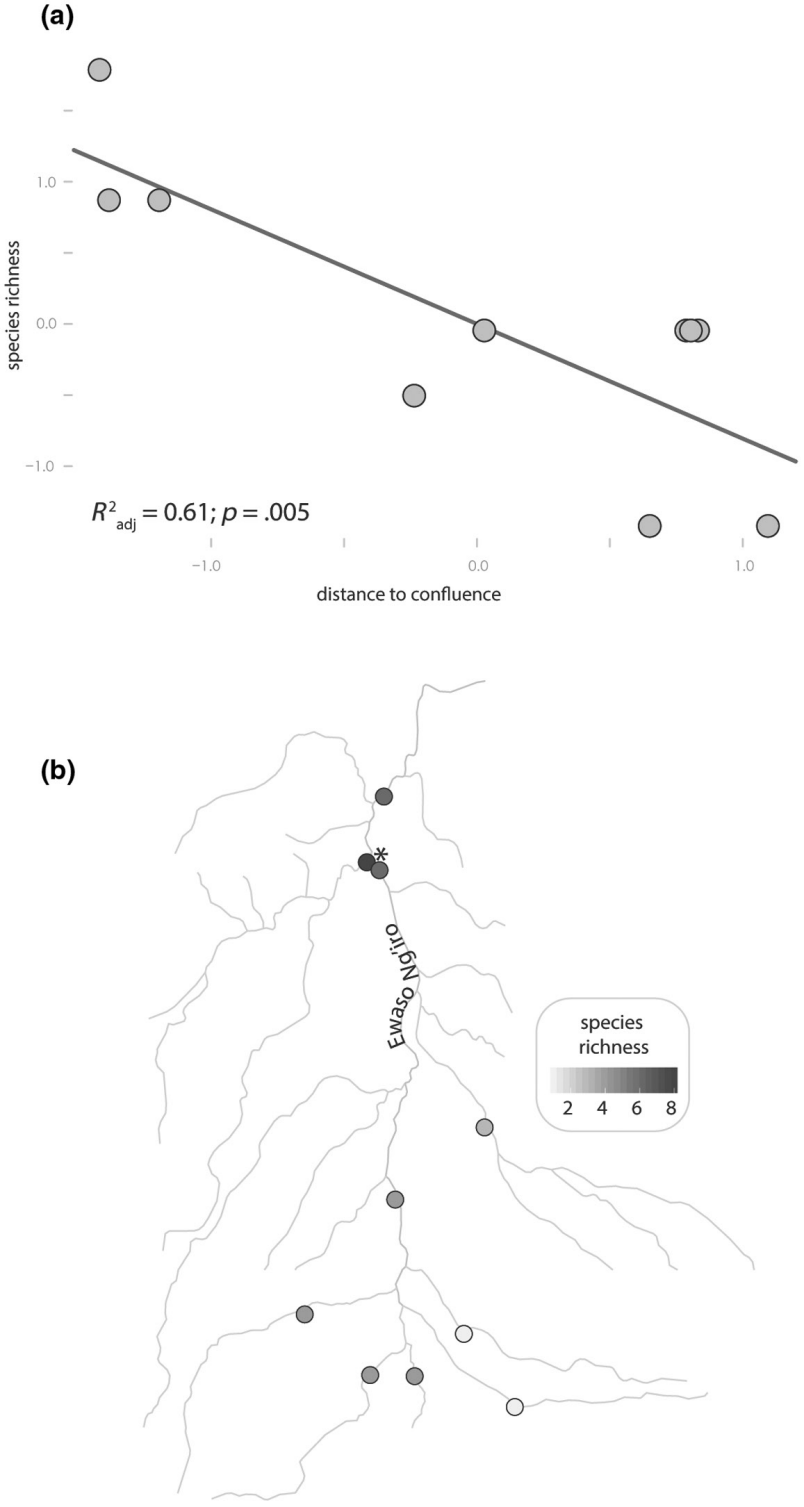
Results of the alpha diversity analysis. (a) Results of a linear regression of distance to the confluence of the Ewaso Ng'iro and Narok rivers on species richness (standardized axes). Provided are the *R* ^2^_adj_ and *p* value from the overall regression. (b) A map of species richness values at each sampling site along the longitudinal gradient. Points indicate sampling sites, colored according to species richness and the asterisk indicates the location of the confluence of the Ewaso Ng'iro and Narok rivers.

Sampling events (*n* = 101) from the 10 localities produced 10,039 specimens representing 10 different species (Schmidt et al., 2020). Alpha diversity analysis indicated that the model including distance to confluence as a predictor of species richness was a better model (AIC = 22.85) compared to a null model (AIC = 31.33). We found a strong negative association between species richness and distance to confluence (*β* = −0.05, *p* = .005), which confirmed a longitudinal gradient of decreasing species richness from the mainstem to headwater tributaries (Figure 2a). The highest diversity occurred at the site of Ewaso Narok at Mpala (*n* = 8 species; Figure 2b), and the lowest at Burguret River and Naro Moru River sites (*n* = 1 species). At the latter two sites, the only species sampled across all months was *Garra hindii*, a generalist species tolerant of low DO and eutrophic conditions.

**FIGURE 3:**
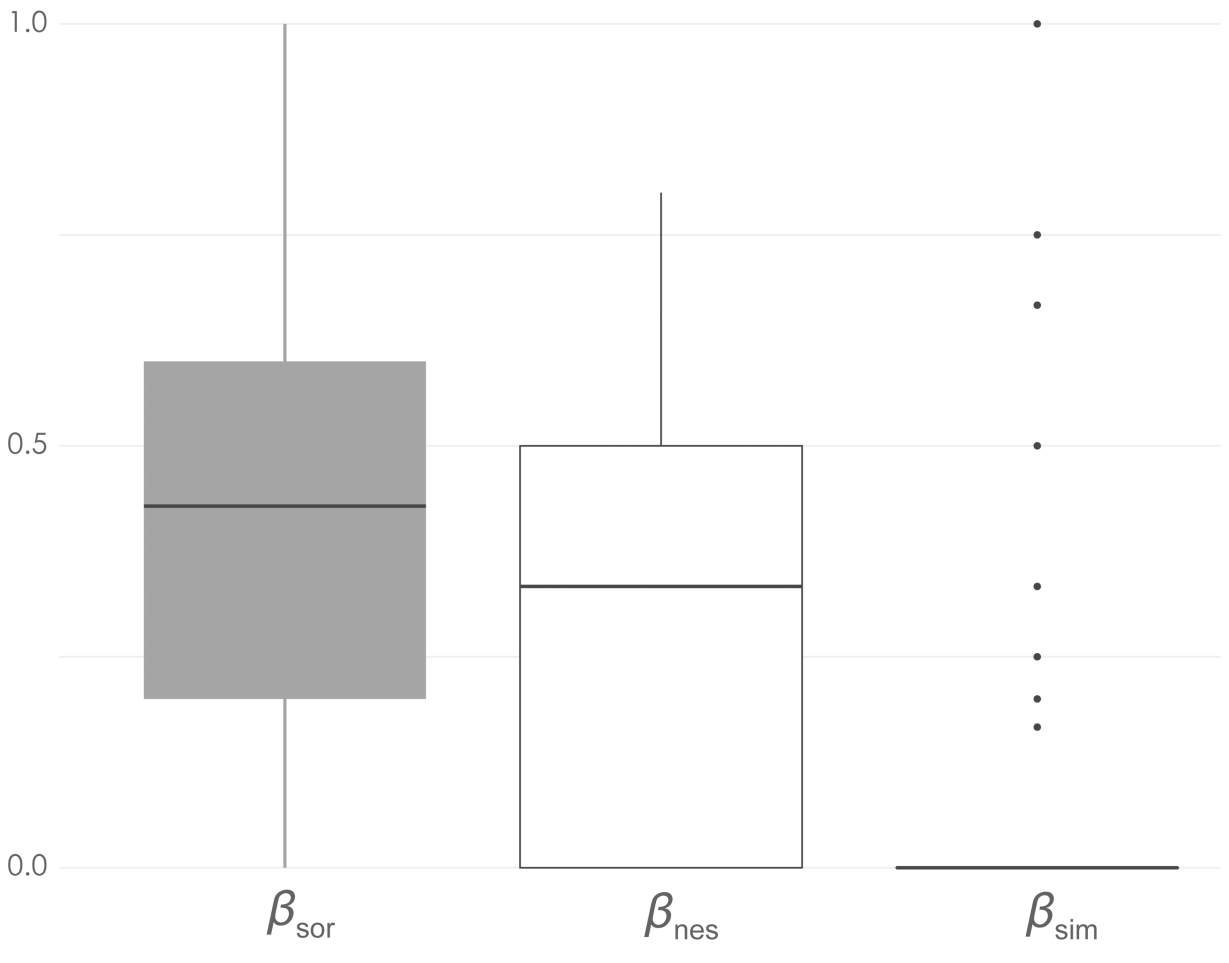
Boxplots of the distribution of values of beta diversity and its components across all sampling events showing total beta diversity (gray boxplot; *β*_sor_), nestedness (*β*_nes_), and turnover (*β*_sim_).

Nestedness was higher (mean *β*_nes_ = 0.30, SD = 0.25) than turnover (mean *β*_sim_ = 0.10, SD = 0.21; *p* < .001; Figure 3). These results indicate beta diversity patterns in this system are driven by stochastic processes (e.g., dispersal and extinction) rather than ecological parameters and we observed a gradient of species losses from species‐rich mainstem sites to species‐poor tributary sites. Temporal beta diversity varied along the longitudinal river gradient with higher variability in species composition among months at mainstem sites, relative to sites farther from the confluence. The one exception to this longitudinal pattern was the Engare Ng'iro site which showed the most variable species composition (mean temporal beta diversity = 0.27). Temporal beta diversity at mainstem sites was highest at Ewaso Ng'iro at Sabuk (mean temporal beta diversity = 0.20), followed by Ewaso Ng'iro at Mpala and Ewaso Narok (mean = 0.19 at both sites) and Nanyuki River (mean = 0.17). Farther upriver, species composition was less variable among months ranging from an average temporal beta diversity of 0.11 at Ngobit River, followed by Ewaso Ng'iro at Ol‐Pejeta (mean = 0.09) and Engare Moyok (mean = 0.05). Burguret River and Naro Moru had no variation in species composition among months (average = 0.00, see above).

**FIGURE 4:**
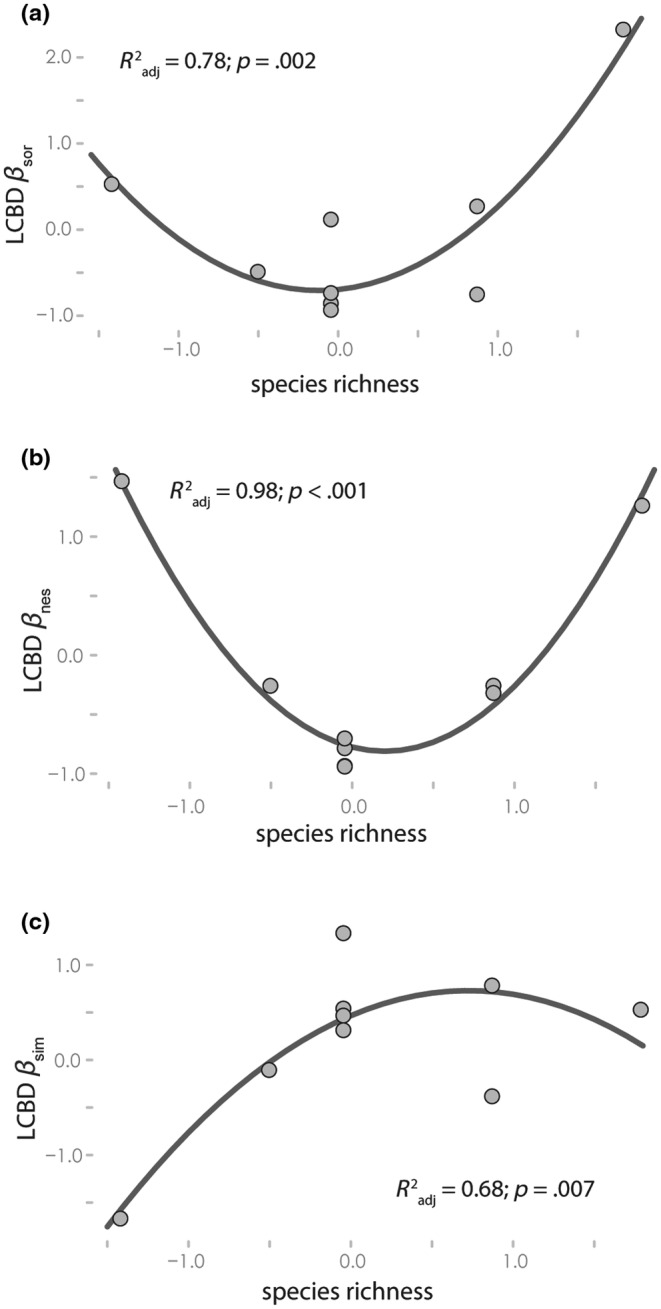
Results of the local contributions to beta diversity (LCBD) analysis assessing effects of species richness on: (a) LCBD to total beta diversity, (b) LCBD to nestedness, and (c) LCBD to turnover. All variables are standardized and the *R* ^2^_adj_ and *p* value from the overall regression are provided in each plot. All 10 sites are shown but in some plots, sites share similar values and points overlap.

Local contributions to beta diversity analysis indicated that a quadratic term for species richness improved model fit, compared to a linear model for *β*_sor_ (delta AIC = 14.96), *β*_nes_ (delta AIC = 39.68), and *β*_sim_ (delta AIC = 6.26). For local contributions to *β*_sor_, we found a significant U‐shaped relationship between LCBD and species richness (*R* ^2^_adj_ = .78; *p* = .002; Figure 4a). Sites with higher LCBD to *β*_sor_ had higher species richness (e.g., Ewaso Narok at Mpala, Ewaso Ng'iro at Sabuk, Ewaso Ng'iro at Mpala), followed by the least biodiverse tributary sites (e.g., Naro Moru and Burguret Rivers). For local contributions to *β*_nes_, model results also indicated a significant U‐shaped relationship between LCBD and species richness (*R* ^2^_adj_ = .98; *p* < .001; Figure 4b). For *β*_sim_, model results showed a significant positive, curvilinear relationship between LCBD and species richness (*R* ^2^_adj_ = .68; *p* = .007; Figure 4c). This relationship appeared to reach an asymptote which may indicate low diversity sites contribute little to turnover in this system, and the relative contribution of a site reaches a limit at mid‐species richness sites (e.g., Ngobit River, Ewaso Ng'iro at Ol‐Pejeta, Engare Ng'iro, Engare Moyok), after which increasing species richness does not increase a site's contribution to turnover.

The db‐RDA analyses indicated environmental and spatial variables determined *β*_sor_, *β*_nes_, and *β*_sim_ in this system but relationships differed between beta diversity components. For *β*_sor_, model selection on environmental factors indicated the mean and standard deviation of stream water temperature were important predictors of total beta diversity; and this reduced model significantly explained variation in total beta diversity within the system (*F* = 3.43, *p* = .001, *R* ^2^_adj_ = .35). Model selection on spatial factors showed that distance to the confluence significantly explained variation in *β*_sor_ (*F* = 3.43, *p* = .001, *R* ^2^_adj_ = .19). For *β*_nes_, model selection on environmental factors indicated the best fit model was a null model, but spatially, longitude significantly explained variation in nestedness (*F* = 3.97, *p* = .016, *R* ^2^_adj_ = .25). For *β*_sim_, model selection on environmental factors indicated the percentage of crop land‐use significantly explained variation in turnover (*F* = 3.72, *p* = .009, *R* ^2^_adj_ = .39) and spatially, latitude significantly explained variation in turnover (*F* = 4.54, *p* = .006, *R* ^2^_adj_ = .46).

**FIGURE 5:**
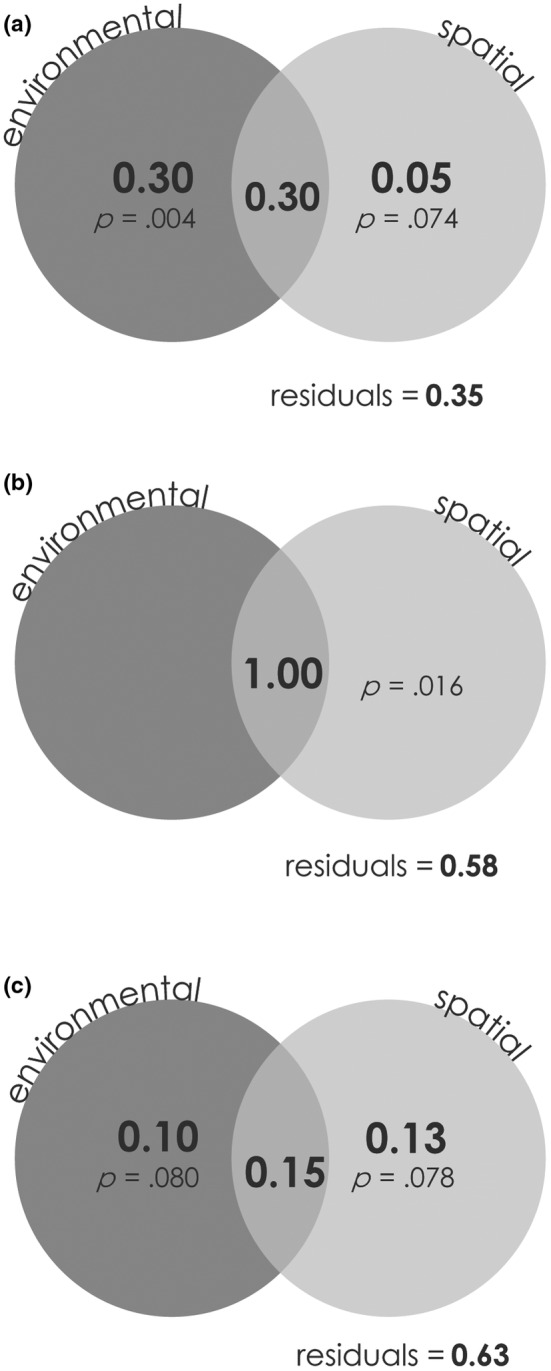
Results of the variance partitioning analysis of effects of environmental (dark gray circles) and spatial (light gray circles) variables on: (a) total beta diversity, (b) nestedness, and (c) turnover. Values represent the *R* ^2^_adj_ (negative values not shot shown) and *p* values for all testable fractions are provided.

For the variation partitioning analysis, environmental and spatial variables explained high percentages of variation in beta diversity metrics, however, few of the effects were significant after accounting for the shared variation between the environmental and spatial factors. For total beta diversity, variation partitioning indicated that environmental and spatial features accounted for 65.0% of variation (*F* = 3.17, *p* = .001; Figure 5a), with 30.0% and 5.0% explained by environmental and spatial predictors, respectively. However, after accounting for shared influences of environment and spatial predictors, only the environmental fraction remained significant (*F* = 2.55, *p* = .004). For nestedness, environmental and spatial predictors explained 41.8% of variation (*F* = 3.966, *p* = .016) but only the spatial effect was still significant after accounting for shared variation (*F* = 3.97, *p* = .016; Figure 5b). For the turnover component, environmental and spatial features accounted for 37.1% of variation (*F* = 4.03, *p* = .003), with 9.9% and 12.6% explained by environmental and spatial predictors, respectively. After accounting for the conditional influences of environmental and spatial variables, neither environmental or spatial factors significantly influenced variation in turnover, independently (Figure 5c).

## 4 DISCUSSION

In this system, the highest biodiversity occurs near the confluence of the Ewaso Ng'iro and Ewaso Narok Rivers and decreases longitudinally from biodiverse mainstem sites to tributary streams. These results are consistent with established longitudinal gradients of increasing species richness from headwaters to mainstem rivers in lotic systems (McGarvey, 2011; McGarvey & Hughes, 2008; Vannote et al., 1980). At two tributary sites, species composition consisted solely of *Garra hindii* across all sampling events. *Garra hindii* appears to consume periphyton, similar to the North American *Campostoma* spp., and seems tolerant of eutrophication which may allow them to persist in degraded environments. Therefore, conditions at the Naro Moru and Burguret Rivers may be restrictive of the presence of less tolerant species in the system. These results are comparable to research from intermittent stream systems where relatively static assemblages in upstream reaches may indicate that harsh conditions allow the persistence of only a limited subset of species (Magalhães et al., 2007).

The relatively less biodiverse communities in the Burguret and Naro Moru rivers compared to the speciose mainstem sites may also suggest a lack of connectivity with the mainstem river because in intermittent streams habitat fragmentation can reduce survival of individuals trapped in isolated pools (Vander Vorste et al., 2020) and prevent recolonization from downstream during flooding events. These two rivers were quite different in the relative condition and habitat of the streams. The Burguret River was sampled below an impoundment created for a flower farm while the Naro Moru River was quite cold (mean temperature 15.3°C) and relatively clean. It is generally assumed that the introduced trout species (e.g., *Oncorhynchus mykiss*) have displaced native fishes in these high elevation Afrotropical streams, but that may not be the case in this river. In the Naro Moru River this may be the limit at which these fishes can persist. During the study, only adult *G. hindii* specimens were collected with no evidence of reproduction occurring in the sampled reach. While *Amphilius grandis* is reported to occur in both the Tana and Northern Ewaso Ng'iro drainages this species is found in higher elevation streams in the Tana drainage than in the Northern Ewaso Ng'iro. Recent studies have also shown that fishes in these Afrotropical higher elevation streams may not be the best indicators of ecosystem health since they are naturally species poor communities (Kleynhans, 1999; Mangadze et al., 2016; Masese et al., 2020; Raburu et al., 2022). Unfortunately, no collections were made at these localities prior to the 1990s so we are unable to determine if the low species richness in these rivers is the natural state or a result of species introductions (i.e., non‐native trout) or land use changes.

The static situation in the Burguret and Naro Moru river localities contrasts with what was observed in the Engare Moyuk. At the beginning of the study, this locality only had two species present, *Garra hindii* and *Clarias gariepinus*. During the El Nino fueled rains in December two other species, *Labeobarbus oxyrhynchus* and *Enteromius paludinosus*, recolonized the site and persisted throughout the duration of the study (Schmidt et al., 2020). The community of fishes in the Engare Ng'iro also varied during the sampling period (mean temporal beta diversity = 0.27) with several species recolonizing the area after rains though not persisting in high numbers (Schmidt et al., 2020). Considering extensive water extraction that occurs in the Upper Ewaso Ng'iro River Basin (Ngigi et al., 2008), this study highlights the need to better understand the potential negative effects on instream connectivity resulting from the expanding agriculture and development in the region.

The community of fishes sampled at the Nanyuki River locality was also quite dynamic during the study. *Garra hindii* are the dominant species at this site throughout the study and present in very high densities (Schmidt et al., 2020). This site on the Nanyuki River was frequently recolonized by other species (e.g., *Amphilius grandis* and *Labeobarbus oxyrhynchus*) after rain events but these species did not persist until the next sampling effort. This locality is downstream from Nanyuki, a town of ~35,000 people, which likely causes diminished water quality favoring the seemingly tolerant *Garra hindii*. Additional surveys are required to better understand the dynamics of the freshwater community in the Nanyuki River and how the anthropogenic changes are affecting it.

Prior analyses of fishes in stream communities found support for both turnover (Lansac‐Tôha et al., 2019; Matthews & Marsh‐Matthews, 2017; Zbinden & Matthews, 2017) and nestedness (Heino, 2011) as the predominant components in beta diversity patterns; however, much of this research derives from perennial streams in temperate or subtropical regions. In intermittent systems, it has been hypothesized that stream drying creates longitudinally nested communities due restricted dispersal or localized extinctions from harsh environmental conditions (Larned et al., 2010). In the Northern Ewaso Ng'iro system, beta diversity patterns indicate a predominance of nestedness in fishes assemblages and may therefore provide support for this hypothesis from understudied tropical systems. Alternatively, these results may also be sensitive to the spatial scale of our analysis. For example, turnover may be the dominant driver of beta diversity patterns with a larger spatial extent (e.g., between basins; Zbinden & Matthews, 2017), rather than the within‐basin extent considered in our study. These results highlight the need for further research on beta diversity patterns in intermittent freshwater systems, especially in tropical systems, to better understand the contexts under which nestedness or turnover patterns may arise.

Contributions of sites to beta diversity within the system indicate that the longitudinal gradient in species richness was an important driver of the contribution of a site to system‐wide beta diversity patterns (Wang et al., 2020). Furthermore, we found that the effects of species richness on LCBD differed considering the beta diversity component considered. For total beta diversity and nestedness, we identified a U‐shaped pattern between species richness and LCBD indicating that sites with both highest and lowest species richness (e.g., mainstem and tributary sites, respectively) show the most ecological uniqueness within this system. For the speciose mainstem sites, this suggests that these sites may represent relatively less impacted communities and are, therefore, potential sites for conservation initiatives (López‐Delgado et al., 2020). On the other hand, the less biodiverse tributary sites may indicate degradation of environmental conditions and, therefore, restoration efforts at these sites may benefit these assemblages (Kong et al., 2017). These potential restoration efforts vary by the sites. For example, in the Nanyuki River it seems fishes are able to recolonize the sampled reach but are not able to persist. Working to decrease water consumption and pollution in the Nanyuki area would likely improve conditions in the stream. In other areas (e.g., the Burguret River) working to remove potential barriers downstream and efforts to decrease water extraction may allow for more recolonization of fishes from mainstem sites. For contributions of sites to the turnover component of beta diversity, we identified a positive, curvilinear relationship between species richness and LCBD which peaked at intermediate species richness. These results may indicate that sites with potentially degraded environmental conditions may be composed of generalist species that are ubiquitous throughout the system, rather than assemblages representing ecologically unique species.

Beta diversity and its components are driven by environmental and spatial factors in this system. For total beta diversity, influences of environmental factors outweighed those of spatial factors and indicated that temperature and temperature variability determine the magnitude of beta diversity. For ectotherms, temperature directly affects organismal physiology and our results may indicate that patterns of beta diversity in this system are driven by environmental filtering on species thermal limits and breaths (Sunday et al., 2019). Alternatively, effects of temperature may be indirectly mediated through variables which covary along temperature gradients. Future studies could incorporate species traits to identify potential intrinsic responses to temperature which may help disentangle direct and indirect effects of temperature on aquatic biodiversity. As previously noted, we are unsure if the relatively depauperate conditions in the Naro Moru are a result of the inability of species to recolonize the reach or because the temperature and elevation at the site represents the physiological limit at which these species can persist. We found that nestedness contributions to beta diversity are spatially structured by the distance to the confluence of the Ewaso Ng'iro and Narok Rivers, indicating species loss from biodiverse mainstem sites to less speciose tributary sites. Spatial influences on nestedness may indicate the presence of gradients in physical harshness that occur in intermittent stream systems when reaches become isolated during drying events (Larned et al., 2010). Therefore, unmeasured environmental variables related to anthropogenic disturbance may cause progressive loss of less tolerant taxa along the longitudinal gradient toward communities dominated by generalists (Gutiérrez‐Cánovas et al., 2013). One possibility is that increasing drought stress from water extraction in headwater streams may progressively reduce regional species pools toward communities of drought tolerant, generalist species (Aspin et al., 2018).

## 5 CONCLUSION AND CONSERVATION IMPLICATIONS

Our results indicate that the areas near the confluence of the Ewaso Narok and Ewaso Ng'iro are the most speciose in this system and are important contributors to the beta diversity. However, these communities of fishes are under immediate threat of inundation from the reservoir resulting from the approved dam downstream of the confluence. While the resulting reservoir may favor some species, the altered flow and temperature regime will likely negatively affect the movement of fishes in the system both upstream and downstream of the impoundment (Bunn & Arthington, 2002). An additional threat to preserving streamflow connectivity in this system is the growth of agriculture and water extraction, especially in tributary sites. Therefore, it will be important to determine whether the effects of anthropogenic stressors on beta diversity patterns are affecting freshwater ecosystem functioning (Fugère, Jacobsen, et al., 2018; McIntyre et al., 2007; Mori et al., 2018) and even the physiological processes of the inhabitants (Fugère, Mehner, & Chapman, 2018) in these areas.

Our results provide preliminary support for hypotheses proposed for intermittent stream systems but more research in this area is needed to indicate whether our findings are generalizable to other intermittent tropical systems. Future studies should continue to investigate beta diversity patterns in tropical montane freshwater systems as many of these systems harbor great biodiversity that may be disproportionately at risk compared to well‐studied temperate regions (Encalada et al., 2019). In addition to biodiversity studies, it is also important to expand traits‐based research efforts and monitoring of abiotic conditions (e.g., streamflow time‐series) in these systems. These datasets will be essential to understanding how stream communities are impacted by the multiple natural and anthropogenic stressors that affect these systems.

## AUTHOR CONTRIBUTIONS

**Ray C. Schmidt:** Conceptualization (lead); data curation (equal); formal analysis (supporting); funding acquisition (lead); investigation (lead); methodology (lead); project administration (lead); visualization (lead); writing – original draft (equal); writing – review and editing (equal). **Taylor Woods:** Data curation (equal); formal analysis (lead); writing – original draft (equal); writing – review and editing (equal). **Wanja D. Nyingi:** Conceptualization (supporting); investigation (supporting); project administration (supporting); writing – original draft (supporting); writing – review and editing (equal).

## FUNDING INFORMATION

Smithsonian Institution Mpala postdoctoral fellowship to RCS funded the bulk of this research. Additional support and resources are provided by the National Museums of Kenya.

## CONFLICT OF INTEREST

None.

## Supporting information


Tables S1‐S2.

## Data Availability

Data from this study are available in the Freshwater Biodiversity Data Portal http://data.freshwaterbiodiversity.eu/metadb/bf_mdb_view.php?entryID=FWM_28.
