# Supplementary material for: Drivers of species richness and beta diversity of fishes in an Afrotropical intermittent river system
Source: Ecol Evol. 2022 Dec 28;12(12):e9659. doi: 10.1002/ece3.9659 (PMC9797352; doi:10.1002/ece3.9659)
Supplement: Supplementary file 1 — Tables S1‐S2. [file ECE3-12-e9659-s001.docx]

**Table S1.** Sites sampled in the Ewaso Ng’iro system, Kenya. For each site, the latitude, longitude, elevation, and species richness are provided.

| **Site** | **Lat** | **Long** | **Elev (m)** | **Richness** |
| --- | --- | --- | --- | --- |
| Burguret River | -0.083 | 36.985 | 1847 | 1 |
| Engare Moyuk | -0.137 | 36.925 | 1847 | 4 |
| Engare Ng’iro | -0.136 | 36.868 | 1871 | 5 |
| Ewaso Narok - Mpala | 0.526 | 36.863 | 1571 | 8 |
| Ewaso Ng’iro - Mpala | 0.516 | 36.867 | 1571 | 6 |
| Ewaso Ng’iro - Ol Pejeta | 0.091 | 36.900 | 1763 | 5 |
| Ewaso Ng’iro - Sabuk | 0.611 | 36.886 | 1486 | 6 |
| Nanyuki River | 0.184 | 37.012 | 1754 | 3 |
| Naro Moru River | -0.177 | 37.054 | 2045 | 1 |
| Ngobit River | -0.057 | 36.783 | 1885 | 4 |

**Table S2.** Predictor variables to explain patterns in beta diversity. For each variable, the class of variable (environmental [env] or spatial [spat]) is provided with a description.

| **Variable** | **Class** | **Description** |
| --- | --- | --- |
| % crop | Env | Percentage of crop land use within a 200 m buffer of the site |
| Distance to confluence | Spat | River distance from the site to the confluence of the Ewaso Narok and Ng’iro Rivers |
| DO | Env | Mean dissolved oxygen |
| pH | Env | Mean pH |
| % pool | Env | Mean percentage of pool habitat within the site |
| % shrub | Env | Percentage of shrub land cover within a 200 m buffer of the site |
| Avg temp | Env | Average temperature |
| SD temp | Env | SD of temperature across months |
| Latitude | Spat | Site latitude |
| Longitude | Spat | Site longitude |
